# Attitudes to smoking cessation and triggers to relapse among Chinese male smokers

**DOI:** 10.1186/1471-2458-6-65

**Published:** 2006-03-14

**Authors:** Tingzhong Yang, K John Fisher, Fuzhong Li, Brian G Danaher

**Affiliations:** 1 Department of Social Medicine, School of Medicine, University of Zhejiang, 353 Yan'an Road, Hangzhou, Zhejiang 310031, China; 2 Oregon Research Institute, Eugene, USA

## Abstract

**Background:**

Smoking is related to many diseases, and the relapse to smoking after cessation in China is noticeable. We examined the attitudes of Chinese male smokers regarding smoking cessation and reasons for relapse.

**Methods:**

We interviewed 201 male smokers in Hangzhou City, Zhejiang province, China who had tried to quit smoking at least once in order to identify reasons for quitting and situations triggering relapse.

**Results:**

The most significant reported reasons for quitting included personal health (77.1%), the cost of cigarettes (53.7%), and family pressures to quit (29.9%). The most common factors triggering relapse were social situations (34.3%), feeling negative or down (13.4%) and times of being alone (8.4%).

**Conclusion:**

Health and family concerns, personal factors, the influence of others and a lack of cessation resources were cited as salient factors concerning smoking cessation among male smokers in this study. Effective smoking control efforts in China will require attention to these influences if China is to curb its current smoking epidemic.

## Background

With an estimated 300 million male smokers and 20 million female smokers, China has more smokers than any other country worldwide, and as a result smoking related diseases are becoming epidemic [1-4]. A growing number of Chinese studies have confirmed that cigarette smoking is the key behavioural risk factor in the incidence of cancer, cardiovascular disease, and COPD (Chronic Obstructive Pulmonary Disease) [5-8]. For example, the prevalence of COPD in China is estimated to exceed 3%, or 25 million people, of whom 72% were smokers [7]. Because of China's large population and the substantial contribution smoking makes to mortality, a number of researchers have argued that more effective, broadly applied smoking control strategies in China could prevent at least 50 million deaths [4,9,10].

Although few smoking cessation activities have been carried out in China, evidence from the 1996 National Prevalence Survey indicated a smoking prevalence rate for men of 63%, with about 17% reporting that they wanted to quit [10]. However, few Chinese smokers have ever tried to quit (11.7%), and the efficacy of these attempts is very low, with just over 2% of smokers becoming successful quitters [10]. Becoming a permanent ex-smoker depends on many factors including overcoming specific barriers to quitting that involve situational triggers and social cues to smoke that often lead to relapse [11-13].

In light of the predominance of male compared to female smokers in China, and the relatively low proportion of successful quitters evidenced in recent surveys, we felt the need to explore current attitudes of Chinese male smokers towards smoking cessation and their reasons for relapse through a representative sample of married men from a large city in China. This information begins to address to the lack of available information on factors related to cessation and relapse among Chinese smokers, and may serve as a basis for the development of future smoking cessation programs.

## Methods

Using a research protocol that was approved by the Department of Social Medicine at the Zhejiang University, face-to-face interviews about smoking cessation behavior were conducted by the first author (TY) and his staff of trained medical students in the homes of married men in Hangzhou City, capital of Zhejiang province in Eastern China. Male smokers were chosen because of the very high smoking rates among men over the age of 20 in China compared to women [10,14]. In addition, contact information for married men was readily accessible from city-identified households and we wished to identify the influence of family support for quitting.

Households were identified by a multi-stage sampling procedure using a city-identified list of family households. First, two city-defined residential districts were chosen with high density family households. Second, from each city-defined neighbourhood area within these districts, blocks of apartment buildings were randomly selected using "Jiedao" (a sub-district neighbourhood administration). Third, from these apartment buildings, household units were randomly sampled by proportional sampling methods, using the serial numbers for each apartment unit. Interviews were only sought from married men from these units who were current smokers ("Is there a smoker in this family who has smoked every day for the last 6 months?"); if none were present, no interview took place. In rare cases where there was more than one male married smoker in the household, a coin was flipped to determine who was to be interviewed. Trained research staff obtained an informed consent agreement from each smoker as a prerequisite for the interview. Interviews were conducted in private in a separate room and took only 30 minutes to complete. A reliability coefficient of 0.96 was obtained when a random 10% of the survey responses were checked by telephone.

This process identified two samples: a) smokers who had never tried to quit, and b) smokers who had tried to quit and relapsed. While all these respondents completed face-to-face interviews, the attempted quitters provided additional data related to situations and reasons for relapse. The interview survey [see Additional file 1] included items tapping the following themes: a)socio-demographics; b) current smoking status; c) smoking history; d) number of past quit attempts and length of abstinence; e) reasons for quitting; and f) situations that triggered relapse. From the responses we were able to rank reasons for quitting and relapse according to frequency.

## Results

Responses were obtained from 95% of those households contacted. Of the 1,922 eligible participants identified and called, 56 individuals refused to be interviewed, thus yielding a final sample of 1,866 participants, of which 906 identified themselves as current smokers. Based upon these results, we calculated an estimated smoking prevalence rate among our total sample of male smokers as 47.1% (906/1922). Of this sample, 203 (22.4%) indicated they had tried to quit smoking at least once in their lifetime ("attempted quitters"), and the remaining 703 smokers indicated that they had never tried to quit. There was no significant difference between attempted quitters versus those who had not tried to quit in terms of age, years of education, occupation, smoking behaviors, duration of smoking, amount smoked, and thoughts about quitting. Nor were there any between-group differences in terms of self-efficacy for quitting ("how would you describe your self-confidence in successfully quitting smoking") and perceived barriers to quitting. The two groups naturally differed only in their quitting intentions, with the percent wanting to quit (24.9%) being significantly lower among those who made no attempt to quit (703/906 = 77.6%)

In Table 1 we include characteristics of our study sample. Complete data were obtained from 201 attempted quitters. The majority started smoking after age 20, and almost 65% smoked 10 or more cigarettes per day. Over 76% of respondents reported a low monthly income of less than 1500 RMB (approximately US$186).

Notwithstanding evidence of a strong desire to quit amongst our relapsed smokers (36.3%), or those wanting to try to quit (24.9%), overall feelings of self-efficacy in being able to become an ex-smoker were low, with only 15.1% reporting that they were "certain" that they could quit. Evidence of this lack of self efficacy in remaining an ex-smoker was reflected in an analysis of the duration of the quitting episode. At the most recent attempt to quit, two weeks or less was the maximum smoke free period for half the sample (Table 2).

Primary reasons for attempting to quit were found to be related to health concerns (for self and family), costs, advice or examples of successful quitting from others, and family pressures to quit (see Table 3). About one quarter of respondents cited restrictions due to public places such as the work place and the home as a reason to quit. When asked about their most frequently used methods for quitting (Survey Item #13), respondents indicated that they used will power most frequently (79.6%) followed by medical measures (11.4%), medications (6.9%), and commercial cessation products (4.5%). Medical measures included using remedies such as Chinese traditional medicines (Zhou mai yan ke) rather than nicotine replacements or western drugs. Not one respondent indicated that he had sought help to quit smoking from a medical practitioner.

Participants in the study were asked a series of questions about situations or feelings that influenced or rekindled the desire to smoke during their period of their abstinence (Survey Questions 15–17). Attempted quitters were asked to rank the most influential factor that was a trigger to smoke (i.e., a temptation to slip). This influence was not necessarily a *cause *to light up or begin smoking again (Question 15). Major triggers to smoke were being in a social situation that involved other smokers (30.8%), following a meal (14.4%), or feeling negative or down (13.4%) (see Table 4).

From a range of items respondents were asked what kinds of situations caused them to relapse (Survey Question 16). Social situations (often involving other smokers) was highest on the list of frequent responses (34.3%), but after a meal (7.5%) was less important than being alone (8.4%), and high or low emotional states (13.4% respectively) (see Table 5).

When asked to identify only *one factor *that caused them to relapse (Survey Question 17), respondents indicated that personal factors such as low perceived sense of self-control (40.3%), the influence of other smokers (28.9%), and a lack of available cessation methods (16.9%) were the most frequent responses.

## Discussion

This report describes results from a household survey of tobacco cessation and relapse related attitudes and behaviors from a representative sample of male smokers living in Hangzhou, an urban city in eastern China. The regional variation in tobacco use in China has been documented [10] with prevalence rates in rural areas higher than the national average for men smokers (68.4% versus 64%) [14,15]. However, in eastern China where our study was conducted, smoking rates are lower than in other areas, and we feel much of our data is representative of this area of China. For example, a recent survey by Yang in 2002 [16] found that the smoking rate for men in Hangzhou was 42.9%. Further, we wish to point out that the WHO definition of smoking (daily smoking for 6 months) used in our survey differs from the 1996 National Survey (smoking at least one cigarette daily)[10]. However, given these differences, our data is similar to National Survey data in terms of the relatively late uptake of smoking after age 20 for most men, the high smoking rates, the proportion who have tried to quit (27% versus 34%) and the low rate of successful lifetime quitters (2.3% nationally versus 0%) in our study.

Our survey interviews identified similar reasons for relapse among our sample of Chinese male smokers to those that have been reported for the broad category of addictive behaviors [17] and smoking cessation [13] in western societies. For example, we identified that relapse was associated with feelings of low self-control, negative stress, times of high elation, and social situations involving other smokers. In addition, we found few differences between triggers or cues to smoke, and situations that caused a person to relapse fully. The relatively short periods of abstinence reported by our respondents underscores the difficulties faced by smokers who want to quit in China. While the absence of available cessation methods was cited as a reason for relapse by only 17% of our sample, it was more likely related to situations where high positive feelings were associated with social situations (e.g., winning at cards). From our data, it would appear that social situations and peer influences are important barriers to smoking cessation in this Chinese population.

Perhaps most central to Chinese culture is the value of family to the individual as well as to Chinese society. Family values emphasize the collective quality in the nature of the individual's life and behaviour, and a strong sense of obligation and responsibility to one's family is cherished as a virtue [18]. In this context, smoking may be perceived as a threat to the health and financial concerns of a family. Yang and Miao [19] recognized the important role of family in designing an effective smoking cessation program based on spouse support. Family and peer influences have been found to be related to smoking in China [18,20,21]. Advice about quitting and examples from others outside the family was cited as third most influential reason to attempt to quit in this study. This evidence suggests that home and family support as well as social support from others may represent important targets for effective cessation program delivery.

Cost was a frequently reported reason for quitting. Although the absolute price of cigarettes per pack is much cheaper in China than in western countries, the ratio of cigarette expense to a smoker's income is much higher. A study by Guindon, Tobin & Yach [22] identified that China was on the list of countries they compared in lowest affordability of cigarettes, after adjusting for purchasing power and exchange rates. But regional economic conditions greatly influence the affordability of cigarettes in China. For example, according to the above study [22], the minutes of labour required to purchase a packet of Marlboro in mainland Shanghai (62 minutes), is almost 3 times that for smokers in Hong Kong (27 minutes), and 6 times for smokers across the China Sea in Taiwan (11 minutes) Thus, cigarette smoking may appear to present a considerable financial burden to smokers. However, considering that the national smoking rate is over 60%, and, that in China cigarettes are cheap, readily available in packs, or sold individually almost everywhere, cost may not actually represent such a significant burden for smokers, particularly in times of recent economic growth.

Chinese culture also places high value on individual "personal power" and considers this as perhaps the most important personal quality, compared to western values, which emphasize appearance, money, and/or prestige among a host of valued personal attributes [23,24]. Handling social interaction and personal relationships well is extremely important and is a foundation of the commercial enterprise and business acumen so endemic in Chinese society. Traditionally, offering people cigarettes has been a custom to enhance personal friendship and relationships, and Chinese people have traditionally shared cigarettes with each other and treated guests with cigarettes. With the rapid rates of economic development in China during the last 20 years, business relationships and social contacts have considerably increased the rates of smoking [2,25,26]. One study reported the most important reasons for smoking in China are embedded in social interactions and personal relationships [27]. This places a large burden on individual self-efficacy for quitting, and refusing offers and temptations to slip within a pro-smoking social milieu.

By regarding public smoking as socially acceptable or desirable, the social milieu in China contains considerable barriers to the promotion of effective smoking cessation opportunities. In addition to being tobacco consumers, China is one of the world's largest producers of tobacco products so that tobacco is widely available [26]. The low desire to quit among smokers suggests a stronger public health agenda to focus more upon making smoking socially unnecessary and/or unacceptable [27]. While at the outset this may appear a forlorn utopian dream, evaluations from western style intervention programs with Chinese youth have shown promise and potential [28]. In addition, cessation efforts targeted at parents and adult smokers could help to reduce the adoption of smoking among future generations [29].

## Conclusion

The results of this study help to fill a void in the literature with regard to identifying important psychosocial issues related to cessation and smoking relapse among male Chinese smokers in a large city. Notwithstanding the relatively encouraging finding that a modest proportion of smokers have a desire to quit, the high rate of recidivism is cause for concern. By highlighting the salient challenges facing attempted quitters in China both from a situational/cultural perspective as well as on a personal level, our data may provide important information for the design of smoking cessation programs and/or the settings in which they might be delivered. Our respondents identified a lack of cessation resources available for well-intentioned quitters, but this explanation may have masked a more personal sense of failure to exercise sufficient will power at a moment of social pressure to light up. In addition, the perceived lack of cessation resources may have been due to a paucity of tax supported public health agencies and environmental support systems (e.g., cigarette taxes, smoke free ordinances) for smoking cessation in China. Compared to most western societies where public health promotion initiatives and revenue from tobacco sales have provided educational, fiscal and regulatory support to curb smoking attitudes and behaviours, public health efforts in Chinese society have not yet succeeded in setting a social agenda that supports the reduction of tobacco use.

## Competing interests

The author(s) declare that they have no competing interests.

## Authors' contributions

TY conceived of the study, conceptualized ideas, supervised its conduct, and designed and coordinated the data collection phase. KF, FL, and BD all helped to interpret findings and contributed to the text. All authors reviewed drafts of the manuscript and approved the version to be published.

## Pre-publication history

The pre-publication history for this paper can be accessed here:



## Supplementary Material

Additional File 1 Relapse Survey. A copy of the survey instrument (translated into English) is presented as a Microsoft Word document. Click here for file

## Figures and Tables

**Table 1:** Demographic and smoking characteristics of relapsed male smokers

Characteristic	% of sample (N = 201)
Age	
< 30 years	14.9
30 – 39 years	35.8
40 – 49 years	31.8
50 years and older	17.5
Education	
Completed elementary school	5.5
Completed junior high & high school	70.6
College and above	23.9
Occupation	
Management	16.9
Science/teaching	12.9
Enterprise clerk	44.3
Commercial clerk	20.9
Other	5.0
Individual Income (RMB) *	
< 1000	41.9
1000 – 1499	34.9
1500 – 1999	8.0
2000 – 2999	5.6
3000 – 4999	3.5
5000 -	6.1
Age of smoking initiation	
< 20 years	43.2
20 – 29 years	30.9
30 years and older	25.9
Cigarettes smoked per day	
< 10 cigarettes	35.3
10 – 19 cigarettes	46.8
20 or more cigarettes	17.9

* 8.05 RMB ~ US$1.

**Table 2:** Length of abstinence in the most recent quit attempt

	N	% of sample (N = 201)	95% C.I.
< 2 weeks	57	28.4	22.3 – 35.1
2 weeks	42	20.9	15.5 – 27.2
1 month	31	15.4	10.7 – 21.2
2 months	15	7.5	4.2 – 12.0
3 months	16	7.9	4.6 – 12.5
4 months	40	19.9	14.6 – 26.1
Total	201	100.0	

**Table 3:** Reasons given for attempting to quit

	N *	% of sample (N = 201)	95% C.I.
Health concern (for self and family)	155	77.1	70.7 – 82.7
Cost	108	53.7	46.6 – 60.8
Family pressure	60	29.9	23.6 – 36.7
Advice and example from others	72	35.8	29.2 – 42.9
Restrictions on smoking (in workplace, on public transportation, at home)	53	26.4	20.4 – 33.0
Social stigma	45	22.4	16.8 – 28.8

* Respondents could indicate multiple answers to this item.

**Table 4:** Most influential trigger to smoke during a period of abstinence

	N	% of sample (N = 201)	95% C.I.
In social situations (e.g., in the company of other smokers, etc)	62	30.8	24.5 – 37.7
After a meal	29	14.4	9.9 – 20.1
When feeling negative, or down	27	13.4	9.0 – 18.9
When feeling positive, or elated	22	10.9	7.0 – 16.1
When feeling stressed	18	9.0	5.4 – 13.8
When feeling tired	12	6.0	3.1 – 10.2
During entertainment (Playing cards for money, playing Majiang, watching sport, etc.)	10	5.0	2.4 – 8.9
While reading or writing	9	4.5	2.1 – 8.3
When alone	7	3.5	1.4 – 7.0
In the presence of alcohol	4	2.0	0.5 – 5.0

**Table 5:** Situations causing relapse among Chinese male smokers

	N	% of sample (N = 201)	95% C.I.
In social situations (e.g., in the company of other smokers, etc)	69	34.3	27.7 – 41.3
When feeling negative, or down	27	13.4	9.0 – 18.9
When feeling positive, or elated	27	13.4	9.0 – 18.9
When alone	17	8.4	5.0 – 13.2
After a meal	15	7.4	4.2 – 12.0
During entertainment (Playing cards for money, playing Majiang, watching sport, etc.)	12	5.9	3.1 – 10.1
While reading or writing	11	5.4	2.7 – 9.5
When feeling tired	10	4.9	2.4 – 8.9
When feeling stressed	9	4.4	2.0 – 8.3
In the presence of alcohol	4	1.9	0.5 – 5.0
