# Supplementary material for: Attitudes to smoking cessation and triggers to relapse among Chinese male smokers
Source: BMC Public Health. 2006 Mar 14;6:65. doi: 10.1186/1471-2458-6-65 (PMC1431522; doi:10.1186/1471-2458-6-65)
Supplement: Additional File 1 — Relapse Survey. A copy of the survey instrument (translated into English) is presented as a Microsoft Word document. [file 1471-2458-6-65-S1.doc]

**Smoking Survey Questionnaire**

Please answer each of the following items as they apply to you. Answer as honestly as you can. Thank you so much.

**Screen**

1. Do you have somebody currently smoke cigarettes (smoke every day for 6 months ) in your family?

[1] Yes [2] No 🡪 exit

The person is male or female?

[1] male [2] female 🡪 exit

1. Is he married?

[1] Yes [2] No 🡪 exit

**Interview Questions**

1. Your name___________________
2. Your age ? (“√” one box)

[1] < 30 years

[2] 30–39years

[3] 40–49years

[4] 50 and over years

1. What is your highest level of education? (“√” one answer)

[1] Elementary school and lower

[2] Junior school

[3] High school

[4] College and over

1. What is your occupation? (“√” one box)

[1] Management

[2] Science/teaching

[3] Enterprise clerk

[4] Commercial clerk

[5] Other (please specify)

1. How much is your income in every month (RMB) in present?

[1] Less than 1000

[2] 1000–1499

[3] 1500–1999

[4] 2000–2499

[5] 2500–2999

[6] 3000–3499

[7] 3500–3999

[8] 4000–4499

[9] 4500–4999

[10] 5000–4999

[11] 6000–6999

[12] 7000–7999

[13] 8000 or more

1. How long have you smoked regularly?

[1] Less than 2 years

[2] 2–3 years

[3] 3–4 years

[4] 4–5 years

[5] 5 or more years

1. On average, how many cigarettes per day do you currently smoke?

[1] Less than 10 cigarettes

[2] 10–19 cigarettes

[3] 20 or more cigarettes

1. Age you started smoking?

[1] Under 20 years

[2] 20–29 years

[3] 30 years and older

1. Have you ever made an attempt to stop smoking?

[1] Yes (Continue)

[2] No (Skip to Question 18)

1. How many times have you ever tried to stop smoking?

[1] 1 time

[2] 2 times

[3] 3 times

[4] 4 times

[5] 5 times

[6] 6 times and over

1. On your most recent quit attempt, how long were you able to stop smoking?
   (“√” one answer)

[1] < 1 week

[2] 1–2 weeks

[3] 2–3 weeks

[4] 3 weeks – 1 month

[5] 1–2 months

[6] 2–3 months

[7] 3–4 months

[8] 4–5 months

[9] 5–6 months

[10] 6 or more months

1. Your reasons for trying to stop smoking? (“√” more than one answer)

[1] Family pressure

[2] Health concern (for self and family members)

[3] Advice and example from others

[4] Cost

[5] Restrictions on smoking in workplace, on public transportation, at home

[6] Social stigma

[7] Other (please specify)

1. Method most often used to quit smoking?

[1] Will power

[2] Behavior approach (stay away from smokers, distract, drink tea and so on)

[3] Medical measures (like nicotine replacement, Chinese traditional medicine)

[4] Family help

[5] Commercial cessation products

[6] Other (please specify)

1. In the above which method do you think was the most effective?_________
2. Most influential trigger to smoke when you tried to quit smoking? (“√” one answer)

[1] In social situations (e.g., in the company of other smokers, etc)

[2] When feeling stressed

[3] When feeling negative, or down

[4] When feeling positive, or elated

[5] During entertainment (playing cards for money, playing Majiang, watching sport, etc)

[6] While reading or writing

[7] When alone

[8] In the presence of alcohol

[9] After a meal

[10] When feeling tired

[11] Other (please specify)

1. The most influential situation that caused your relapse? (“√” one answer)

[1] In social situations (e.g., in the company of other smokers, etc)

[2] When feeling stressed

[3] When feeling negative, or down

[4] When feeling positive, or elated

[5] During entertainment (playing cards for money, playing Majiang, watching sport, etc)

[6] While reading or writing

[7] When alone

[8] In the presence of alcohol

[9] After a meal

[10] When feeling tired

[11] Other (please specify)

1. Which one most caused your relapse?

[1] Low self- control

[2] The influence of other smokers

[3] A lack of available cessation methods

[4] Little family support

[5] Other (please specify)

1. Do you want to quit smoking?

[1] Don’t want to quit

[2] Want to attempt to quit smoking

[3] Strong desire to quit smoking

1. If you try to quit smoking, how would you describe your self-confidence in successfully quitting smoking?
    (“√” one answer)

[1] Will be successful

[2] May be successful

[3] May succeed or fail

[4] Likely to fail

Home address:

Home telephone number:

Interviewer:

Date:

Subject signature:

Checker:

Date:
